# Additive Manufacturing of Regorafenib Tablets: Formulation Strategies and Characterization for Colorectal Cancer

**DOI:** 10.3390/polym17172302

**Published:** 2025-08-26

**Authors:** Fatemeh Safari, Azin Goudarzi, Hossein Abolghasemi, Hussein Abdelamir Mohammad, Mohammad Akrami, Saeid Mohammadi, Ismaeil Haririan

**Affiliations:** 1 School of Chemical Engineering, Alborz Campus, University of Tehran, Tehran 1417935840, Iran; 2 Department of Biotechnology & Pharmaceutical Engineering, School of Chemical Engineering, College of Engineering, University of Tehran, Tehran 1417935840, Iran; 3 Center for Separation Processes Modeling and Nano-Computations, School of Chemical Engineering, College of Engineering, University of Tehran, Tehran 1417935840, Iran; 4 Department of Pharmaceutics, College of Pharmacy, University of Al-Qadisiyah, Al Diwaniyah 58002, Iraq; 5 Department of Pharmaceutical Biomaterials and Medical Biomaterials Research Center, Faculty of Pharmacy, Tehran University of Medical Sciences, Tehran 1417614411, Iran; 6 Department of Pharmacology and Toxicology, Faculty of Pharmacy, International Campus, Tehran University of Medical Sciences, Tehran 1417614411, Iran; 7 Department of Pharmaceutics, Faculty of Pharmacy, Tehran University of Medical Sciences, Tehran 1417614411, Iran

**Keywords:** 3D printing, regorafenib, release, tablet, colorectal cancer, oral delivery, kinetic

## Abstract

Significant efforts have been dedicated to developing controlled-release systems for the effective management of colorectal cancer. In this study, a once-daily, delayed-release regorafenib (REG) tablet was fabricated using 3D printing technology for the treatment of colorectal cancer. For this, a hydrogel containing 80 mg of the drug in a matrix of hyaluronic acid, carboxymethyl cellulose, Pluronic F127, and glycerol was prepared to incorporate into the shell cavity of tablet via a pressure-assisted microsyringe (PAM). The shell was printed from an optimized ink formulation of Soluplus^®^, Eudragit^®^ RS-100, corn starch 1500, propylene glycol 4000, and talc through melt extrusion-based 3D printing. In vitro release assays showed a drug release rate of 91.1% in the phosphate buffer medium at 8 h and only 8.5% in the acidic medium. Drug release kinetics followed a first-order model. The results showed smooth and uniform layers based on scanning electron microscopy (SEM) and drug stability at 135 °C upon TGA. FTIR analysis confirmed the absence of undesired covalent interactions between the materials. Weight variation and assay results complied with USP standards. Mechanical strength testing revealed a Young’s modulus of 5.18 MPa for the tablets. Overall, these findings demonstrate that 3D printing technology enables the precise fabrication of delayed-release REG tablets, offering controlled-release kinetics and accurate dosing tailored for patients in intensive care units.

## 1. Introduction

Colorectal cancer (CRC) is known as a global health problem, hence the critical necessity of prevention, prognosis, and appropriate treatment for improving the quality of life while reducing mortality [1]. According to a WHO report, colorectal cancer is known not only as the third most widespread cancer, but also the second cause of cancer-related deaths among all cancers screened in populations. It also estimated that this type of cancer accounts for about one in ten cancer cases.

Colorectal cancer is typically initiated from the appearance of proliferated cell mass, polyps, in the inner lining of colon and rectal. Upon further genetic transformation of the colonic epithelium of the benign polys, this abnormal cell growth will result in cancerous cells [2]. In this regard, different challenges have been reported for the management of metastatic colorectal cancer [3]. Systemic therapy has been considered as a primary approach for treating this metastatic cancer. Chemotherapy (single or combination regimens), targeted therapy, immunotherapy, surgery, radiation and ablation techniques are other strategies for controlling the disease, according to cancer stages [4]. However, upon disease progression and exhaustion of standard therapies, alternative treatments become essential for patients [5].

REG, an FDA-approved drug, is considered for the treatment of colorectal cancer when the cancer has progressed or if no effective standard therapies are available, which is known as salvage therapy [6]. This small molecule inhibits not only oncogenic protein kinases and tyrosine kinase receptors like KIT and RET, but also platelet-derived growth factor receptors (PDGFRs), vascular endothelial growth factor receptors (VEGFRs), and fibroblast growth factor receptors (FGFRs) [7]. Through this multi-targeted inhibition, it suppresses cancer cell proliferation, neovascularization, tumor progression, and oncogenesis.

Furthermore, REG has been administered in combination with TAS-102 (trifluridine/tipiracil) to enhance treatment efficacy for metastatic colorectal cancer following failure of other therapies, resulting in improved overall survival during later-line treatment [8]. REG is classified as a Biopharmaceutics Classification System (BCS) II drug, characterized by poor aqueous solubility but high permeability. Due to its low solubility, specific formulation strategies are essential to enhance its bioavailability including cyclodextrin complexation, polymer-based nanoparticles, solid dispersions, supercritical CO_2_ impregnation, use of cosolvents, and others [9,10].

In addition, to treat patients with cancer disease under pretreated conditions, REG is medicated at a total daily dose of 160 mg by taking four 40 mg tablets for the first 3 weeks of a month. Converting this multi-dose regimen into a once-daily, extended-release tablet could significantly improve patient compliance by reducing missed doses. Moreover, dose adjustments are often required during treatment, with REG doses ranging from 80 mg to 160 mg at initiation and during dose escalation or reduction phases to optimize therapeutic outcomes and manage tolerability [11].

Recently, 3D printing technology has been increasingly utilized in pharmaceutical manufacturing to meet evolving therapeutic needs [12]. This approach not only enables customization and cost-effective production but also facilitates the creation of complex dosage forms and polypills, supporting localized and on-demand manufacturing [13]. Indeed, additive manufacturing has provided tailored dosage for personalized medicine [14]. Moreover, it allows precise manipulation of solid dosage forms to achieve desired drug release kinetics [13]. Previous studies have demonstrated that 3D printing effectively facilitates the fabrication of extended- and sustained-release tablets [12].

The melt extrusion process has been integrated with 3D printing techniques, particularly fused deposition modeling (FDM), to improve the fabrication of tablets with tailored drug release profiles [15]. Various release kinetics—including sustained, delayed, and immediate release—can be achieved in pharmaceutical solid dosage forms by employing hot melt extrusion (HME) to disperse drug substances uniformly within excipients [16]. Soluplus^®^, a water-soluble triblock copolymer, has been widely employed in hot melt extrusion (HME) to improve the solubility of poorly water-soluble drugs [17]. Additionally, polymers from the Eudragit^®^ family, known for their thermoplastic and melt-extrudable properties, are extensively used in the development of colonic drug delivery systems [18]. However, the melt extrusion process is unsuitable for thermolabile drugs. To address this limitation, recent approaches have introduced core–shell platforms for 3D-printed tablets, wherein thermo-sensitive drug substances are encapsulated within a hydrogel-based core, which is then surrounded by a melt-extruded shell [19,20].

Hydrogels as biocompatible and biodegradable drug delivery systems (DDSs) have garnered significant interest in pharmaceutical formulations [21,22]. Some oral-targeted DDSs have been developed to achieve delayed drug release at desired sites beyond the gastrointestinal tract (GI), wherein the drug-loaded hydrogel is incorporated within the core of a hard gelatin capsule [23].

This hydrogel-based core not only provides biocompatible, versatile and stimuli responsive carrier but also enables controlled-release kinetics.

Drug-loaded hydrogels provide drug delivery platforms that improve efficacy and safety compared to traditional formulation [24]. Various polymers and materials have been employed in hydrogel preparation for this purpose. Notably, poloxamers such as Pluronic F-127, approved by the Food and Drug Administration (FDA), are widely used due to their biocompatibility, excellent wetting properties, and versatile physicochemical characteristics [25]. However, a notable limitation of Pluronic F-127 is its insufficient gel strength [26]. Incorporating hydrophilic polymers such as hyaluronic acid (HA), as well as chitosan and cellulose derivatives, into poloxamer hydrogels can improve their rheological strength, viscosity, bioadhesiveness, and ability to provide controlled drug release [25].

This study aims to develop an oral sustained-release system for targeted delivery of REG to manage colorectal cancer. To this end, core–shell tablets were fabricated using an extrusion-based 3D printing technique, wherein a REG-loaded hydrogel was extruded into the core compartment and encapsulated by a shell produced via melt extrusion of Soluplus^®^, Eudragit^®^ RS-100, and other excipients. The resulting 3D-printed tablets were physicochemically characterized to ensure compliance with United States Pharmacopeia (USP) standards.

## 2. Materials and Methods

### 2.1. Materials

The active ingredient, REG, was obtained from Roshd Parsian Pharmaceutical Technologies Company (Tehran, Iran). The inactive ingredients were sourced as follows: Soluplus^®^ from BASF (Florham Park, NJ, USA), talc from Sigma Co. (Darmstadt, Germany), polyethylene glycol 4000 and carboxymethyl cellulose from Kimigaran Emrooz Company (Tehran, Iran), sodium lauryl sulfate from Merck (Darmstadt, Germany), corn starch 1500 from Iran Daru Company (Tehran, Iran), Eudragit^®^ RS-100 from Rohm Pharma Polymers Company (Kyoto, Japan), and Pluronic F127 from Bio Basic Company (Markham, ON, Canada). All materials provided in pharmaceutical grade. All solvents in analytical grade, including methanol, ethanol, and glycerol, were purchased from Merck.

### 2.2. Tablet Design

**Figure 1:**
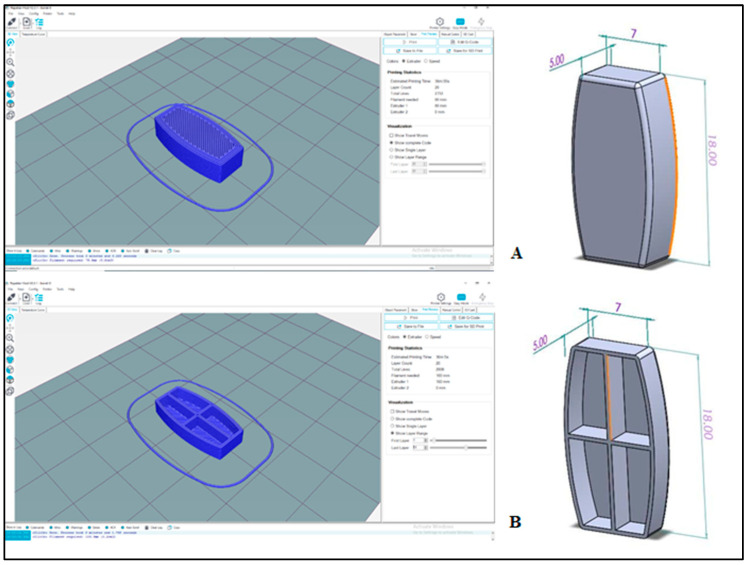
View of the REG Tablet design as displayed by Repetier (**left**) and SolidWorks (**right**) softwares: (**A**) outer layout and (**B**) internal design.

By modeling commercially available tablets using SolidWorks software (version 2022), a barrel-shaped tablet was designed with the following dimensions: 18 mm longitudinal diameter, 9 mm middle transverse diameter, 7 mm marginal transverse diameter, and 5 mm thickness. The final tablet design was created in SolidWorks and subsequently imported into Repetier software (version 2023), as illustrated in Figure 1.

### 2.3. Formulation of the REG Tablet

Since the melting point of REG (206.0–210.0 °C) is close to the maximum operating temperature of the 3D printer (210 °C), the drug was dissolved in the hydrogel to encapsulate inside the tablet shell.

#### 2.3.1. Core Hydrogel

To prepare the hydrogel, sodium lauryl sulfate (1%) was first dissolved in deionized water at 4–8 °C. Pluronic F127 was then added and the mixture was stirred for 1 h. Next, hyaluronic acid was incorporated and mixed under the same conditions for 24 h. Separately, REG was levigated in glycerin before being added to the previous solution and thoroughly mixed. During levigation, sedimentation of hydrophobic REG particles may occur due to poor wetting by the levigating vehicle. This can be prevented by increasing the amount of vehicle and adding wetting agents such as glycerol and SLS. To increase the viscosity of the poloxamer hydrogel and ensure it is syringeable, a specific amount of carboxymethyl cellulose was added to the mixture.

#### 2.3.2. 3D-Printed Shell

To fabricate the shell compartment, various ratios of Eudragit^®^ RS-100, Soluplus^®^, polyethylene glycol 4000, talc, and corn starch 1500 were thoroughly mixed and loaded into the melt extruder of the 3D bioprinter (OmidAfarinan Mohandesi Ayandeh Co., Tehran, Iran). After melting, the extrudability and printability of the resulting filament were evaluated to select the optimal formulation.

### 2.4. Printing Process

The 3D bioprinter operates mainly by extrusion-based printing, using either pneumatic actuators to deposit inks layer-by-layer. It supports multiple printheads (2 or 4) for printing diverse biomaterials.

The tablet design was processed using Repetier slicing software (version 2.2.4) and printed with a 3D printer instrument equipped with two independent nozzles. The shell materials were extruded through a 0.4 mm diameter nozzle at 135 °C, with a printing speed of 100 mm/s, a bed temperature of 50 °C, and under a pressure of 2 atmospheres. Simultaneously, the drug-loaded hydrogel was injected into four designated pits using the second nozzle, a Pressure-Assisted Microsyringe (PAM) extruder, to ensure even hydrogel distribution during printing. Finally, the internal space of the tablet was sealed by printing the upper lid.

### 2.5. Characterization

#### 2.5.1. Tablet Morphology

The morphology of the printed tablets was examined using scanning electron microscopy (SEM) by VAGA Tescan instrument (Brno, Czech Republic). Samples were analyzed at 15 kV and at magnifications of ×4 and ×60 to assess the dimensions of the printed layers. Prior to imaging, the samples were coated with a thin layer of gold in a vacuum chamber to enhance electron conductivity.

#### 2.5.2. Fourier-Transform Infrared (FTIR) Spectroscopy

To find out the chemical properties of the materials used in printing the REG tablet, 5 mg of each material was first mixed with 300 mg of potassium bromide, then 150 mg of the mixture was placed in a matrix cylinder with a pressure of 80 psi. Finally, the spectra were achieved by PerkinElmer instrument (Springfield, IL, USA) upon scanning between wavelengths of 400 to 4000 cm^−1^ to identify the various vibrations and chemical interactions.

#### 2.5.3. Thermogravimetric Analysis (TGA)

For this analysis, approximately 22.5 mg of the powdered tablet sample was subjected to LINSEIS TGA PT1600 instrument (Robbinsville, NJ, USA). The measurement was performed from room temperature to 600 °C at a heating rate of 20 °C/min under a nitrogen atmosphere.

#### 2.5.4. Assay and Dissolution Tests

UV–Vis spectrophotometry of Jasco, UV-1500 (Tokyo, Japan) was used to determine REG concentrations at 261 nm. To prepare the standard stock solution, 4 mg of pure REG was dissolved in 50 mL of a water:methanol mixture (90:10) and sonicated for 5 min. A standard solution with a concentration of 53.3 μg/mL was then prepared using phosphate buffer (pH 6.8) as the diluent and measured in triplicate for absorbance. For assay determination, ten tablets were ground, and a quantity equivalent to the mean tablet weight was transferred to a 100 mL volumetric flask. After sonication and volume adjustment, the solution was centrifuged for 5 min, and the absorbance of the supernatant was measured at 261 nm. The unknown sample concentration was calculated by comparing its absorbance to that of the standard solution, enabling the assay of the 3D-printed tablets.

An in vitro dissolution test was performed to evaluate the release profile of REG. The test was conducted in 250 mL of acidic medium (0.1 M hydrochloric acid, pH 1.2) for 1 h, followed by phosphate buffer (pH 6.8) for 7 h. Sodium lauryl sulfate (0.1%) was added to maintain sink conditions. Dissolution was carried out using Apparatus I at 100 rpm and 37 °C. Samples were collected at specified intervals from 30 min to 8 h and diluted six-fold before absorbance measurement. The REG standard solution for the calibration curve was prepared by dissolving 5 mg of pure REG in 50 mL of solvent, sonicated for 5 min to yield a 100 μg/mL stock solution, and further diluted to obtain standards of 6.25, 12.5, 25, 50, and 75 μg/mL. The amount of REG released at each time point was determined by UV–Vis spectrophotometry at 261 nm in triplicate.

To analyze the drug release behavior of the 3D-printed REG tablet, dissolution data were fitted to various kinetic models, including zero-order, first-order, Higuchi, and Korsmeyer–Peppas models.

#### 2.5.5. Weight Variation

Ten tablets were weighed individually using a calibrated balance, and the relative standard deviation was calculated according to USP guidelines to determine weight variation.

#### 2.5.6. Mechanical Test

Young’s modulus was calculated from the stress–strain curve obtained during mechanical testing of 3D-printed tablets (dimensions: 18 cm × 10 cm × 5 cm) using a Universal Tester (Mechanical Strength Tester Model Z050, Zwick Roll, Kennesaw, GA, USA), in accordance with USP <1217>. Each tablet was placed between parallel, smooth jaws, and compression was applied along the longitudinal axis at a speed of 20 mm/min until the sample was crushed.

## 3. Results and Discussion

Regorafenib, a drug substance with a chemical formula of C_21_H_15_ClF_4_N_4_O_3_ in its base form, a molecular weight of 482.82, and a LogP value ranging from 4.49 to 4.53, is insoluble in water, indicating its lipophilicity. Regorafenib is moderately thermostable within pharmaceutical formulations under controlled conditions but sensitive to high heat and humidity.

The core–shell REG tablet was fabricated using extrusion-based additive manufacturing, in which a drug-containing hydrogel was encapsulated within a printed polymeric shell. This approach improves solubility of REG, enables customized dosing, and allows for tailored drug release kinetics, facilitating targeted delivery to colorectal cancer.

### 3.1. Tablet Fabrication

In this study, separate series of formulations were developed for the hydrogel core and the shell compartment to fabricate an 80 mg REG tablet. As shown in Table 1, formulations F1 to F5 represent different hydrogel compositions, each containing 80 mg of REG with varying ingredient ratios. In core compartment, polymers are chosen due to their ability to form a biocompatible hydrogel that can encapsulate the active REG. For hydrogel preparation (Table 1), a constant amount of hyaluronic acid (HA) was used to leverage its beneficial properties, including hygroscopicity and immunomodulatory effects [27]. Additionally, HA is known to improve intestinal barrier function and reduce inflammation in inflammatory diseases [28]. CMC, like HA, provides a hydrophilic, gel-forming matrix with good mucoadhesion, which may aid controlled release and localized delivery. Pluronic F127 is a thermoresponsive polymer that forms micelles and gels at body temperature, helping in sustaining drug release. Glycerol acts as a co-solvent for poorly soluble REG. Given the high lipophilicity of REG, it was initially levigated in glycerin (F1) before being added to a Pluronic F127 solution, which was prepared by stirring for 24 h at 4 °C. To address the drug sedimentation observed in F1, the amount of glycerin was doubled and 10 mg of SLS was added in F2, resulting in reduced sedimentation. Complete resolution of sedimentation was achieved in F3 by doubling the concentrations of both SLS and Pluronic F127 compared to the previous formulation. However, the resulting hydrogel was too fluid; therefore, 4% CMC was incorporated in F4 to increase viscosity and improve syringeability. It was noted that higher concentrations of CMC led to syringe blockage.

For the formulation of the tablet shell, a combination of Soluplus^®^ and Eudragit^®^ RS-100 as the primary thermoplastic polymers, along with PEG 4000 as a plasticizer, was used to optimize extrudability, printability, and non-floating properties. Soluplus^®^, a hydrophilic polymer, enhances both the solubility and extrudability of the materials during the melting process. Eudragit^®^ RS-100 as a pH-independent polymer was incorporated to provide a controlled barrier for REG release. The shell is made from an “optimized ink formulation” suitable for melt extrusion 3D printing, requiring polymers that provide mechanical strength, printability, and controlled drug release. Propylene glycol 4000 may act as a plasticizer, improving flexibility. The shell protects the drug-containing hydrogel and controls the release profile (delayed release) of REG.

As shown in Table 2, increasing the ratio of Soluplus^®^ to Eudragit^®^ RS-100 from S1 to S2 improved the extrudability of the resulting filament. The addition of corn starch in S3 further enhanced the fluidity of the mixture, resulting in a smoother filament. Finally, the issue of buoyancy was addressed by incorporating an appropriate amount of talc into the formulation (S4, S5).

### 3.2. Results of Characterization

#### 3.2.1. Dimensions and Morphology

**Figure 2:**
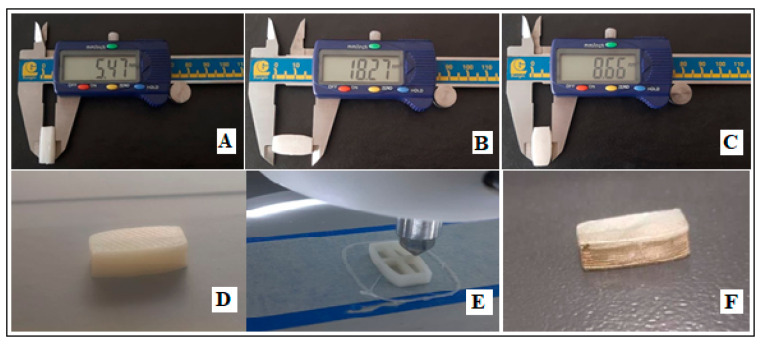
3D-printed REG tablet. (**A**–**C**) Measurement of tablet dimensions using a caliper; (**D**) fabricated tablet; (**E**) printing process; (**F**) gold-coated tablet prepared for SEM analysis.

The thickness, length, and width of the tablet were measured using a digital caliper from BAKER Gauges, DDC30 model (Pune, India) as shown in Figure 2. The mean accuracy for thickness, as well as middle transverse and longitudinal diameters, of the tablets were about 105%, 101%, and 98.2%, respectively. The differences between SolidWorks tablet design dimensions and the actual printed tablet dimensions primarily arise due to material shrinkage/warping, printer resolution, filament/extrusion inconsistencies, printer calibration limits, and design features being below the printer’s printable thresholds. For example, this difference can be attributed to the lower resolution of layers printed by nozzle diameters of 0.4 µm rather than smaller diameter size. In comparison, the dimensions of hard gelatin capsules available in the market depend on formulation dose requirements, including cap length from about 6.2 to 12.95 mm and body length from 9.3 to 22.2 mm. The 3D-printed tablet of REG with dimensions of about 18 × 9 mm is close to capsule size of 2.

Figure 2D displays the fabricated 3D-printed tablet, while Figure 2E illustrates the printing process and the internal pits designed for hydrogel filling.

**Figure 3:**
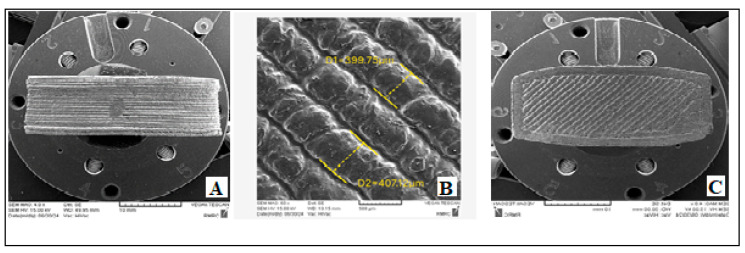
SEM micrographs of the 3D-printed tablet: (**A**) side view; (**B**) magnified view of the layers; (**C**) bottom view.

The morphology of the printed tablet was analyzed using SEM imaging. As shown in Figure 3, the printed layers appear regular and smooth. In some areas, however, layer overlap is observed, which may be attributed to solvent evaporation from the polymer. According to the micrographs, the average filament diameter was approximately 400 μm, consistent with the nozzle diameter.

#### 3.2.2. FTIR Analysis

**Figure 4:**
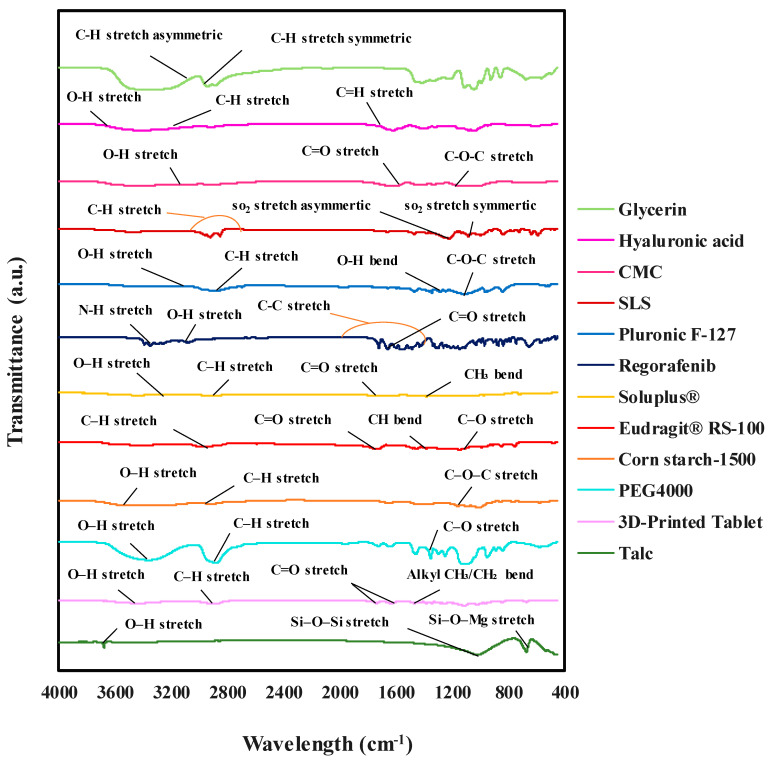
FTIR spectra for 3D-printed tablet of REG and its individual components.

Figure 4 presents the FTIR spectrum of the REG tablet in comparison with the spectra of its individual components. This analysis was perform to identify and confirm the chemical identity and purity of the components and possible interactions.

The FTIR spectrum of REG shows a peak at 3358 cm^−1^ corresponding to N-H stretching, while absorption bands at 3111 cm^−1^ and 1657 cm^−1^ are attributed to the O-H and C=O stretching, respectively. Additional peaks at 1468, 1544, and 1658 cm^−1^ are associated with aromatic ring stretching vibrations. For Pluronic F-127, a prominent peak at 2887.8 cm^−1^ is observed, corresponding to C–H stretching vibrations. Absorption bands at 1343.87 cm^−1^ and 1110.82 cm^−1^ are related to O–H bending and C–O–C stretching, respectively. The broad band at 3466.21 cm^−1^ is due to hydroxyl group vibrations. Hyaluronic acid is characterized by an absorption band at 1620 cm^−1^ (C=O stretching), a band at 2924 cm^−1^ (C–H vibration), and a peak at 3410.2 cm^−1^ (O–H stretching). The FTIR spectrum of glycerin features a broad peak between 3200 and 3500 cm^−1^, attributed to O–H stretching vibrations, with additional bands at 2882.7 cm^−1^ and 2937 cm^−1^ corresponding to symmetric and asymmetric C–H stretching. In the sodium lauryl sulfate spectrum, absorption peaks at 1219.4 cm^−1^ and 1084.4 cm^−1^ represent the symmetric and asymmetric stretching vibrations of the SO_2_ group, respectively. Peaks at 2851, 2919, and 2958 cm^−1^ are associated with hydrocarbon group stretching, while peaks below 900 cm^−1^ correspond to C–C vibrations. Carboxymethyl cellulose exhibits a peak at 3429.91 cm^−1^ (O–H stretching), with absorption bands at 1017.49 cm^−1^ and 1633.28 cm^−1^ corresponding to ether and carboxyl group stretching, respectively.

For the tablet shell ingredients, the Eudragit^®^ RS-100 spectrum shows an absorption peak at 2953.40 cm^−1^ (C–H stretching), a peak at 1732.20 cm^−1^ (C=O stretching), and a peak at 1146.39 cm^−1^ (C–O stretching). The absorption peak at 3438 cm^−1^ is attributed to carboxylic hydroxyls, and the peak at 1463 cm^−1^ to –CH bending vibrations. The FTIR spectrum of Soluplus^®^ features peaks at 3441.63 cm^−1^ (O–H stretching), 2925.10 cm^−1^ (C–H stretching), 1632.36 cm^−1^ and 1742.20 cm^−1^ (C=O stretching in amide groups), and 1447.79 cm^−1^ (CH_3_ bending). In the talc spectrum, the Si–O–Si stretching vibration appears at 1016.18 cm^−1^, Si–O–Mg stretching at 670 cm^−1^, and O–H stretching at 3677.45 cm^−1^. Corn starch 1500 shows characteristic absorption peaks at 3414.09 cm^−1^ (O–H stretching), 2928.64 cm^−1^ (C–H stretching), and 1158.63 cm^−1^ (C–O–C stretching). PEG 4000 exhibits prominent peaks at 1351.71 cm^−1^ (C–O stretching), 3368.28 cm^−1^ (O–H stretching), and 2873 cm^−1^ (C–H stretching). The FTIR spectrum of the 3D-printed tablet, indicated by a black dashed line in Figure 4, shows a peak at 3423.21 cm^−1^, which corresponds to O–H stretching vibrations from carboxymethyl cellulose, PEG, glycerin, and hyaluronic acid. The peak at 2891.53 cm^−1^ is attributed to C–H stretching vibrations of the tablet components. Peaks at 1736.29 cm^−1^ and 1638.9 cm^−1^ are assigned to carbonyl group vibrations in carboxymethyl cellulose and hyaluronic acid, while the peak at 1467.67 cm^−1^ corresponds to the bending vibrations of alkyl groups such as CH_3_ or CH_2_. Overall, the FTIR spectrum of the 3D-printed tablet aligns with the spectra of the individual components, with some absorption bands shifted due to hydrogen bond interactions.

#### 3.2.3. TGA Thermal Analysis

**Figure 5:**
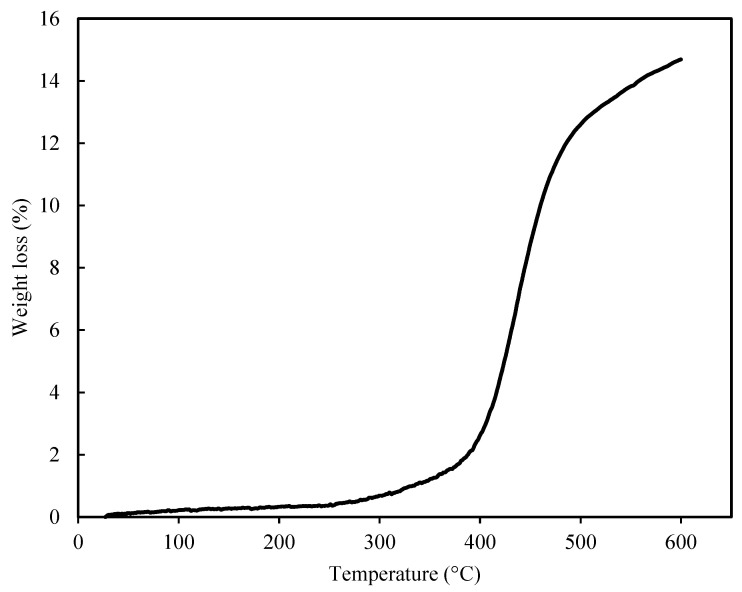
TGA curve illustrating the thermal behavior of the 3D-printed REG tablet during heating up to 600 °C.

The results of the thermal analysis for 3D-printed tablets containing REG, heated up to 600 °C, are presented in Figure 5. As shown, the tablets exhibit a partial weight loss of approximately 2.17% up to 392 °C, which can be attributed to solvent evaporation. Additionally, a further weight loss of up to 14.69% at 559.8 °C corresponds to material decomposition. Importantly, no thermal decomposition was observed within the temperature range used for 3D printing (below 150 °C), confirming that the printing process occurs under safe thermal conditions for tablet fabrication.

#### 3.2.4. Weight Variation Results

According to the USP standard <2091>, the acceptable weight variation for tablets weighing more than 324 mg is 5%. The weight variation results for ten printed tablets are shown in Table S1. As indicated, the printed tablets exhibited a weight deviation of 3.1%, which falls well within the acceptable range.

#### 3.2.5. Assay and Dissolution Profile

The tablet assay results are presented in Table 3. With an assay value of 98.8% for the REG tablet—falling within the USP-specified range of 90% to 110%—the product meets the USP requirements for tablet assay.

The release percentages of the tablet at various time intervals were determined using the calibration curve (Figure S1).

**Figure 6:**
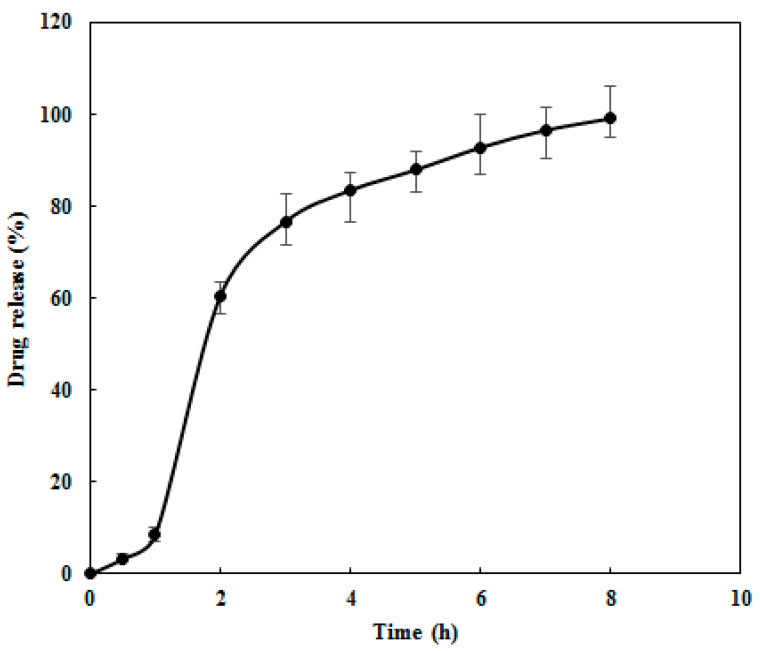
Dissolution profile showing the release of REG from the 3D-printed tablet.

The dissolution testing for the REG tablet was conducted in both acidic (HCl, pH 1.2) and phosphate buffer (pH 6.8) media. Release percentages at various time intervals were calculated using the calibration curve (Figure S1). As shown in the release profile of the core–shell 3D-printed REG tablet (Figure 6), drug release in acidic medium remained below 8.5% within the first hour, meeting the typical USP acceptance criteria for the release amount in acidic media for delayed-release dosage forms. Subsequently, release increased significantly in phosphate buffer, reaching 99.1% ± 2.7 within 8 h. This delayed-release profile addresses concerns about drug instability in the gastric environment [29].

This targeted release is achieved through the deliberate selection of functional polymers: Eudragit^®^ RS-100, which restricts premature drug release due to its low permeability in the gastric environment, and Soluplus^®^, which improves solubility and promotes controlled drug release in the intestinal tract [30,31]. The dissolution profiles indicate that the 3D-printed REG tablets exhibit a delayed-release pattern consistent with a prolonged-release trend, in contrast to the immediate-release profile observed for the commercial REG tablets [32]. This strategy not only enhances patient compliance by enabling once-daily dosing but also offers the advantages over immediate-release formulations including targeted intestinal delivery by reducing gastric exposure, sustained drug release for improving pharmacokinetic profiles, potential decrease in side effects, flexibility in manufacturing by 3D printing, and agreement with quality standards [29]. These benefits support with the purpose of optimizing therapy with REG in colorectal and other cancers.

In comparison, most REG delivery studies in the literature have focused on designing nanoparticle carriers like hyaluronic acid-conjugated liposome, polymeric nanoparticles such as PEGylated PLGA NPs, cyclodextrin complexes, or solid dispersions [33,34].

In contrast to our core–shell 3D-printed tablets, these delivery systems are mostly in injectable forms for systemic delivery, focusing on overcoming REG’s poor solubility and bioavailability, targeting colorectal cancer, and aiming to improve systemic exposure and reduce toxicity, which are essential for clinical outcomes. However, some investigations developed lipid or polymeric nanoparticles for sustained release or enhanced permeability. In a study, REG-loaded PLGA microspheres have been formulated for localized, sustained drug delivery to improve transarterial chemoembolization (TACE) treatment of hepatocellular carcinoma (HCC). Compared to our study, REG release from microspheres took 30 days [35].

PEGylated PLGA nanoparticle formulations have been developed with biphasic delayed release profiles and improved oral bioavailability, showing sustained drug release patterns but generally maintaining gastric resistance similar to our 3D-printed tablet [33].

The marketed REG tablets, such as Stivarga^®^, are immediate release tablets.

Offering superior advantages, our innovative 3D-printed REG with tailored controlled release and thorough USP-compliant physicochemical and mechanical characterization has clinically meaningful release profile matching GI transit. However, in future in vivo pharmacokinetics and bioavailability, scalability and long-term stability should be addressed. The incorporation of bioadhesive hydrogel materials may also enhance mucosal residence time and targeted release, a feature not commonly reported in standard REG tablets.

#### 3.2.6. Drug Release Kinetic Models

The polymer’s physical and chemical properties, including its microstructure, solubility, swelling, erosion behavior, and interaction with the drug, strongly influence the drug release mechanism and thus determine which kinetic model best describes the release profile. In our formulation, polymers of pluronic acid, CMC, Eudragit^®^, and Soluplus^®^ are used as hydrogel or shell components, each having distinct swelling, erosion, and diffusion characteristics that affect drug release rates and mechanisms. For example, hydrophilic polymers like CMC often show diffusion-based release fitting the Higuchi or Korsmeyer–Peppas models, where swelling and diffusion are key mechanisms. Eudragit^®^ polymers, with varying pH solubility profiles, often result in more complex or multi-phase release kinetics that may fit Korsmeyer–Peppas or require combined models, reflecting erosion and diffusion. Pluronic acid and Soluplus^®^, known for solubilizing poorly soluble drugs and forming micellar or gel-like matrices, can influence release kinetics dominated by diffusion or swelling, also aligning often with Higuchi or Korsmeyer–Peppas models.

Accordingly, the experimental data obtained from the dissolution test were analyzed using various mathematical models, including zero-order, first-order, Higuchi, and Korsmeyer–Peppas. These models were applied to identify the best fit that accurately describes the release mechanism of REG from the 3D-printed tablet. In addition to identifying the best fitting model, this comparative approach provides insight into the physicochemical release behavior from the designed core–shell structure.

**Figure 7:**
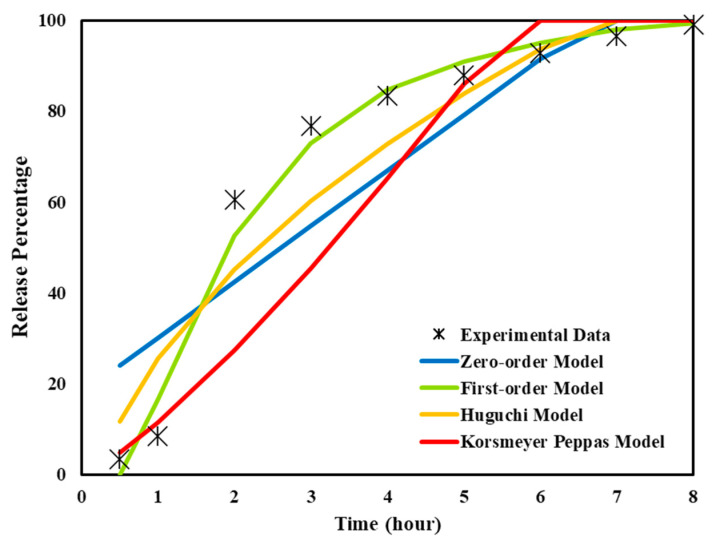
Fitting of experimental drug release data to four kinetic models: zero-order, first-order, Higuchi, and Korsmeyer–Peppas.

Among them, the first-order model was determined to be the most appropriate, exhibiting the highest correlation coefficient (R^2^ = 0.9622) with the equation y = −0.2446x + 2.1654. This indicates an excellent agreement between the model and the experimental data (Table 4; Figure 7), where the fitted curves of all four models are compared to the experimental release profile.

According to the first-order model, as shown in Table 4, the release rate constant (k) was determined to be 0.2449 h^−1^, and the drug release half-life (t_1/2_) was calculated to be approximately 2.83 h. These parameters indicate that the release rate is dependent on the remaining drug concentration, suggesting that drug diffusion or erosion of the carrier matrix primarily governs the release mechanism. This implies that as the drug concentration decreases over time, the release slows down proportionally, which is a characteristic behavior of diffusion-controlled or erosion-mediated systems.

The Higuchi release model was evaluated as the second-best fit, exhibiting a correlation coefficient of R^2^ = 0.89. The linear equation derived from plotting the cumulative amount of drug released against the square root of time is expressed as y = 0.0188x + 0.6171. In this diffusion-based release model, the slope (HK) of 0.0188 represents the drug release rate from the matrix, while the intercept of 0.6171 corresponds to the initial amount of drug released. Despite this, the higher R^2^ value of the first-order model indicates it provides a more accurate description of the release kinetics in this study.

However, it is important to note that the Higuchi model assumes a uniform drug distribution and a constant diffusion gradient across the matrix, which may not entirely apply to the non-homogeneous core–shell structure used in this formulation. Nonetheless, due to its widespread application and its capacity to describe early-stage diffusion behavior, it was retained for comparison.

The Korsmeyer–Peppas model, commonly used to interpret the initial phase (typically up to 60%) of drug release, showed a correlation coefficient of R^2^ = 0.8788 and a release exponent (n) of 1.2469. The value of n suggests a Super Case II transport mechanism, which is typically associated with swelling and polymer chain relaxation. Given that most of the data points fall beyond 60% release, the model may not accurately reflect the full release profile in this study and is potentially subject to overfitting.

The application of multiple kinetic models, despite their assumptions and limitations, enhances understanding of the release behavior from the 3D-printed structure. Among them, the first-order model not only showed the best statistical fit but also mechanistically supports the diffusion-controlled release from the hydrogel core through the polymeric shell.

Our previous study demonstrated the critical role of polymer selection and formulation strategy in modulating drug release profiles. For example, a sustained-release pattern for 3D-printed tacrolimus tablets was achieved by using polyvinyl alcohol as the primary polymer in the shell, combined with hydroxypropyl methylcellulose as a drug-containing gel in the core compartment [13].

Li et al. developed core–shell 3D-printed verapamil hydrochloride tablets to achieve controlled drug release for chronotherapy. A commercial verapamil tablet was encapsulated within a shell fabricated by fused deposition modeling (FDM) 3D printing, using filaments produced via hot melt extrusion (HME) from a hydroxypropyl methylcellulose (HPMC) and polyethylene glycol 400 (PEG 400) mixture. These tablets demonstrated customizable delayed-release profiles ranging from 4 to 8 h, which could be tailored by adjusting the filament composition and shell thickness [36]. Like us, the polymer blends are designed for controlled release using a 3D-printed shell but with a solid core. This tablet showed pulsatile release with programmable lag times 4–8 h (vs. 1 h for our REG tablet). The mechanical strength varies according to filament and shape.

Incorporating Eudragit^®^ L-100 and HPMC HME L-100 polymers in the shell section, along with Kollidon^®^ SR in the core, enabled the fabrication of a 3D-printed mesalamine tablet exhibiting delayed-release kinetics—releasing approximately 5% of the drug within the first 5 h—followed by a sustained release extending up to 24 h, effectively targeting the large intestine [37]. Compared to our formulation, a compressed tablet has been incorporated into the core compartment instead of hydrogel. Moreover, the lag time before drug release is longer than that of our formulation. However, both are delayed-release dosage forms.

Additionally, 3D-printed core–shell propranolol HCl tablets have been developed as a beta-blocker for cardiovascular diseases. In this tablet, a commercial immediate-release tablet in the core is coated with a shell composed of cellulose acetate, mannitol, and PEG through pressure-assisted extrusion. Like our formulation, the delayed-release system delivered drug release from 48% to 84% over 12 h, which was tunable by shell composition. In comparison, our formulation is innovative with a hydrogel core via PAM, contrasting with both verapamil and propranolol studies that use commercial tablets or filaments as cores. Overall, mechanical and regulatory compliance aspects were equivalent and met pharmacopeial standards.

Furthermore, zero-order release kinetics were observed for acetaminophen from 3D-printed tablets, where Kollidon^®^ VA-64 was incorporated in the core, and Eudragit^®^ RS-PO and Eudragit^®^ E-PO polymers were integrated into the shell section of the tablets [38].

In a previous study, ketamine hydrochloride tablets with a core–shell design were developed using melt extrusion-based 3D printing to fabricate the tablet shell, enabling the injection of drug-loaded gel into the tablet cavities [12]. The formulation incorporated Soluplus^®^ and Eudragit^®^ RS-PO polymers in the shell, while carboxymethyl cellulose served as the hydrogel polymer encapsulating ketamine hydrochloride. This design achieved complete (100%) controlled drug release within 12 h.

#### 3.2.7. Mechanical Test Results

**Figure 8:**
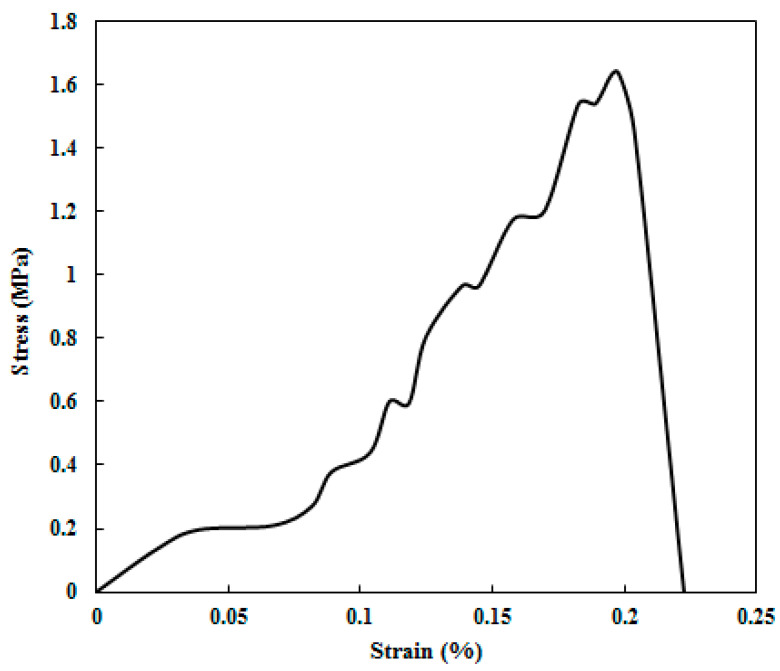
Compressive stress–strain curve of the 3D-printed REG tablet.

Figure 8 presents the stress–strain curve of a 3D-printed REG tablet subjected to compressive force applied perpendicular to its cross-sectional area. The tablet withstands a maximum load of approximately 222.6 N and reaches its breaking point at a strain of about 0.20%. The curve exhibits an almost linear response up to a stress of 0.22 MPa. Following the first yield point at a strain of approximately 0.07, strain hardening occurs. After several minor yield events and subsequent rehardening, the material experiences sudden failure at around 0.20% strain. Young’s modulus was calculated to be 5.18 MPa, while both the compressive strength and fracture strength were measured at 1.65 MPa. The presence of multiple yield points observed in the curve can be attributed to factors such as internal structural characteristics, surface curvature effects, and varying compression mechanisms that have been shown to produce multiple yield points in similar materials [39,40,41].

The impressive load capacity obtained from the mechanical test tablet highlights the tablet’s strength and physical durability during the subsequent pharmaceutical process like blister packaging and transportation [42]. The variation in mechanical strength observed for the 3D-printed REG tablet in this study compared to other reports [15,38,42] can be attributed to differences in formulation parameters, including polymer type, ingredient ratios, internal microstructure of the printed matrix, and printing techniques. For instance, the higher mechanical strength of the REG tablet relative to the 3D-printed ketamine hydrochloride tablet from our previous study is likely due to a lower proportion of Soluplus^®^ in the REG formulation, the inclusion of talc and corn starch-1500 as excipients, and differences in internal cavity design and tablet dimensions [12].

## 4. Conclusions

For the first time, a delayed-release formulation of REG was developed using a core–shell structure fabricated by melt extrusion-based 3D printing. The tablet design features a polymeric shell printed from a blend of polymers, encapsulating a hydrogel containing REG inside the tablet’s internal cavity. Quality control tests including weight variation, drug assay, and dissolution met USP standards, ensuring consistent dosage and performance. SEM imaging showed well-defined, smooth printed layers, indicating good morphological quality. Thermal analysis (TGA) confirmed no drug degradation within the printing temperature range, demonstrating stability during manufacture. Mechanical testing showed the tablets have sufficient strength and durability for pharmaceutical handling and processing. The tablets exhibit a controlled, delayed drug release profile governed by first-order kinetics, effectively enabling targeted intestinal release. This delayed-release design supports convenient once-daily dosing, potentially improving patient compliance and optimizing colorectal cancer therapy. Thus, the study contributes a new application of 3D printing technology to develop controlled-release oral cancer medication with specific dosing and release profiles, which is a technological advancement in pharmaceutical formulation and delivery.

## Figures and Tables

**Table 1:** Hydrogel formulations for REG Tablets.

Formulation *	F1	F2	F3	F4	F5
REG (mg)	80	80	80	80	80
Glycerin (mg)	60	120	120	120	120
CMC (mg)	0	0	0	20	40
Hyaluronic acid (mg)	20	20	20	20	20
Pluronic F-127 (mg)	50	50	100	100	100
SLS (mg)	0	10	20	20	20
No drug sedimentation	×	×	✔	✔	✔
Syringeability	Soupy (×)	Soupy (×)	Soupy (×)	✔	Blockage

* All materials were added in mg. Total volume equals 500 μL. ‘×’ = failure; ‘✔’ = success.

**Table 2:** Formulation of the shell compartment of the 3D-printed tablet with varying ingredient weight percentages.

Formulation	S1	S2	S3	S4	S5
Eudragit^®^ RS-100	45	35	30	20	20
Soluplus^®^	35	45	40	40	40
PEG 4000	20	20	20	20	20
Corn starch-1500	0	0	10	10	5
Talc	0	0	0	10	15
Temperature (°C)	150	150	145	140	135
Printability	×	✔	✔✔	✔✔✔	✔✔✔
Extrudability	×	✔	✔	✔	✔
Non-Floating	×	×	×	✔	✔✔

‘×’ = failure; ‘✔’ = acceptable; ‘✔✔’ = good; ‘✔✔✔’ = excellent.

**Table 3:** Assay results for 3D-printed REG tablet (80 mg).

	Standard	Sample	Concentration (µg/mL)	Assay (%)
Abs1	0.455	0.45	52.7	98.8
Abs2	0.455	0.452	52.9	99.3
Abs3	0.455	0.448	52.5	98.4
Mean	0.455	0.45	52.7	98.8
STD	0.000	0.002	0.234	0.439
RSD	0.000	0.444	0.444	0.450

**Table 4:** R^2^ values and kinetic parameters (k, n, t_1/2_) with standard deviations (SDs) for four models applied to the 3D-printed REG tablet. The first-order model exhibited the best fit to the experimental data.

Model	R^2^	k	n	t_1/2_ (h)	SD (±)
Zero-order	0.7799	12.2927	-	4.07	0.4810
First-order	0.9622	0.2449	-	2.83	0.0212
Higuchi	0.8886	47.2306	-	1.12	1.5285
Korsmeyer–Peppas	0.8788	11.5450	1.2469	0.08	0.0132

## Data Availability

All data were analyzed during this study are included in this published article.

## Supplementary Materials

The following supporting information can be downloaded at https://www.mdpi.com/article/10.3390/polym17172302/s1. Figure S1: Calibration curve for release medium; Table S1: Calculation of the weight variation of the 3D-printed regorafenib tablet.

## References

[1] World Health Organization (2021). Colorectal Cancer. https://www.who.int/news-room/fact-sheets/detail/colorectal-cancer.

[2] Institute N.C. (2025). Genetics of Colorectal Cancer. https://www.cancer.gov/types/colorectal/hp/colorectal-genetics-pdq.

[3] Khan S.Z., Lengyel C.G. (2023). Challenges in the management of colorectal cancer in low- and middle-income countries. Cancer Treat. Res. Commun..

[4] Society A.C. (2023). Treatment for Colorectal Cancer. https://www.cancer.org/cancer/colon-rectal-cancer/treating.html.

[5] UK C.R. (2025). Gerson Therapy. https://www.cancerresearchuk.org/about-cancer/treatment/complementary-alternative-therapies/individual-therapies/gerson.

[6] Sastre J., Argilés G., Benavides M., Feliú J., García-Alfonso P., García-Carbonero R., Grávalos C., Guillén-Ponce C., Martínez-Villacampa M., Pericay C. (2014). Clinical management of regorafenib in the treatment of patients with advanced colorectal cancer. Clin. Transl. Oncol..

[7] Wilhelm S.M., Dumas J., Adnane L., Lynch M., Carter C.A., Schütz G., Thierauch K., Zopf D. (2011). Regorafenib (BAY 73-4506): A new oral multikinase inhibitor of angiogenic, stromal and oncogenic receptor tyrosine kinases with potent preclinical antitumor activity. Int. J. Cancer.

[8] Hsieh M.-C., Rau K.-M., Lin S.-E., Liu K.-W., Chiu C.-C., Chen C.-I., Song L.-C., Chen H.-P. (2022). An observational study of trifluridine/tipiracil-containing regimen versus regorafenib-containing regimen in patients with metastatic colorectal cancer. Front. Oncol..

[9] Bai H., Wang J., Phan C.U., Chen Q., Hu X., Shao G., Zhou J., Lai L., Tang G. (2021). Cyclodextrin-based host-guest complexes loaded with regorafenib for colorectal cancer treatment. Nat. Commun..

[10] Sheikhi A., Hamedi S., Sodeifian G., Razmimanesh F. (2025). Improvement of the dissolution of the antineoplastic drug regorafenib through impregnation into pullulan polysaccharide using supercritical fluid technology: Optimization of the process. J. CO2 Util..

[11] Bekaii-Saab T.S., Ou F.-S., Ahn D.H., Boland P.M., Ciombor K.K., Heying E.N., Dockter T.J., Jacobs N.L., Pasche B.C., Cleary J.M. (2019). Regorafenib dose-optimisation in patients with refractory metastatic colorectal cancer (ReDOS): A randomised, multicentre, open-label, phase 2 study. Lancet Oncol..

[12] Karami T., Ghobadi E., Akrami M., Haririan I. (2024). Fabrication of a controlled-release core-shell floating tablet of ketamine hydrochloride using a 3d printing technique for management of refractory depressions and chronic pain. Polymers.

[13] Abdollahi A., Ansari Z., Akrami M., Haririan I., Dashti-Khavidaki S., Irani M., Kamankesh M., Ghobadi E. (2023). Additive manufacturing of an extended-release tablet of tacrolimus. Materials.

[14] Rastpeiman S., Panahi Z., Akrami M., Haririan I., Asadi M. (2024). Facile fabrication of an extended-release tablet of ticagrelor using three dimensional printing technology. J. Biomed. Mater. Res. Part A.

[15] Wickramasinghe S., Do T., Tran P. (2020). FDM-based 3D printing of polymer and associated composite: A review on mechanical properties, defects and treatments. Polymers.

[16] Bandari S., Nyavanandi D., Dumpa N., Repka M.A. (2021). Coupling hot melt extrusion and fused deposition modeling: Critical properties for successful performance. Adv. Drug Deliv. Rev..

[17] Guembe-Michel N., Nguewa P., González-Gaitano G. (2025). Soluplus^®^-based pharmaceutical formulations: Recent advances in drug delivery and biomedical applications. Int. J. Mol. Sci..

[18] Dos Santos J., da Silva G.S., Velho M.C., Beck R.C.R. (2021). Eudragit^®^: A versatile family of polymers for hot melt extrusion and 3D printing processes in pharmaceutics. Pharmaceutics.

[19] Vigata M., Meinert C., Hutmacher D.W., Bock N. (2020). Hydrogels as Drug Delivery Systems: A Review of Current Characterization and Evaluation Techniques. Pharmaceutics.

[20] Omari S., Ashour E.A., Elkanayati R., Alyahya M., Almutairi M., Repka M.A. (2022). Formulation development of loratadine immediate- release tablets using hot-melt extrusion and 3D printing technology. J. Drug Deliv. Sci. Technol..

[21] Lei L., Bai Y., Qin X., Liu J., Huang W., Lv Q. (2022). Current understanding of hydrogel for drug release and tissue engineering. Gels.

[22] Peppas N.A., Bures P., Leobandung W.S., Ichikawa H. (2000). Hydrogels in pharmaceutical formulations. Eur. J. Pharm. Biopharm..

[23] Bajpai S.K., Bajpai M., Dengre R. (2003). Chemically treated hard gelatin capsules for colon-targeted drug delivery: A novel approach. J. Appl. Polym. Sci..

[24] Buzhor M.G., Abdi F., Luo Z., Leroux J. (2024). Colonic delivery of aqueous suspensions using 3D printed capsules. Adv. Mater. Technol..

[25] Khaliq N.U., Lee J., Kim S., Sung D., Kim H. (2023). Pluronic F-68 and F-127 based nanomedicines for advancing combination cancer therapy. Pharmaceutics.

[26] Coeshott C.M., Smithson S., Verderber E., Samaniego A., Blonder J.M., Rosenthal G.J., Westerink M. (2004). Pluronic^®^ F127-based systemic vaccine delivery systems. Vaccine.

[27] Iaconisi G.N., Lunetti P., Gallo N., Cappello A.R., Fiermonte G., Dolce V., Capobianco L. (2023). Hyaluronic acid: A powerful biomolecule with wide-ranging applications. Int. J. Mol. Sci..

[28] Kotla N.G., Isa I.L.M., Rasala S., Demir S., Singh R., Baby B.V., Swamy S.K., Dockery P., Jala V.R., Rochev Y. (2021). Modulation of gut barrier functions in ulcerative colitis by hyaluronic acid system. Adv. Sci..

[29] Baira S.M., Srinivasulu G., Nimbalkar R., Garg P., Srinivas R., Talluri M.V.N.K. (2017). Characterization of degradation products of regorafenib by LC-QTOF-MS and NMR spectroscopy: Investigation of rearrangement and odd-electron ion formation during collision-induced dissociations under ESI-MS/MS. New J. Chem..

[30] Ding Y., Chang S., Xie Z., Yu D.-G., Liu Y., Shao J. (2020). Core–shell Eudragit S100 nanofibers prepared via triaxial electrospinning to provide a colon-targeted extended drug release. Polymers.

[31] Pignatello R., Corsaro R., Bonaccorso A., Zingale E., Carbone C., Musumeci T. (2022). Soluplus^®^ polymeric nanomicelles improve solubility of BCS-class II drugs. Drug Deliv. Transl. Res..

[32] Sawicki E., Schellens J.H.M., Beijnen J.H., Nuijen B. (2016). Inventory of oral anticancer agents: Pharmaceutical formulation aspects with focus on the solid dispersion technique. Cancer Treat. Rev..

[33] Panigrahi D., Swain S., Sahu P.K., Ghose D., Jena B.R. (2024). Quality by design enabled formulation development of regorafenib monohydrate loaded PEGylated PLGA polymeric nanoparticles: Enhanced oral bioavailability and biopharmaceutical attributes. Nanomed. J..

[34] Shnaikat S.G., Shakya A.K., Bardaweel S.K. (2024). Formulation, development and evaluation of hyaluronic acid-conjugated liposomal nanoparticles loaded with regorafenib and curcumin and their in vitro evaluation on colorectal cancer cell lines. Saudi Pharm. J..

[35] Li X., He G., Su F., Chu Z., Xu L., Zhang Y., Zhou J., Ding Y. (2020). Regorafenib-loaded poly (lactide-co-glycolide) microspheres designed to improve transarterial chemoembolization therapy for hepatocellular carcinoma. Asian J. Pharm. Sci..

[36] Li R., Pan Y., Chen D., Xu X., Yan G., Fan T. (2022). Design, preparation, and in vitro evaluation of core–shell fused deposition modelling 3D-printed verapamil hydrochloride pulsatile tablets. Pharmaceutics.

[37] Alshammari N.D., Almotairy A., Almutairi M., Zhang P., Al Shawakri E., Vemula S.K., Repka M.A. (2024). Colon-targeted 3D-printed mesalamine tablets: Core-shell design and in vitro/ex-vivo evaluation. J. Drug Deliv. Sci. Technol..

[38] Wang H., Vemula S.K., Bandari S., Repka M.A. (2023). Preparation of core-shell controlled release tablets using direct powder extrusion 3D printing techniques. J. Drug Deliv. Sci. Technol..

[39] Davies P.N., Worthington H.E., Podczeck F., Newton J.M. (2007). The determination of the mechanical strength of tablets of different shapes. Eur. J. Pharm. Biopharm..

[40] Castrati L., Mazel V., Diarra H., Busignies V., Tchoreloff P. (2017). Effect of the Curvature of the Punches on the Shape of the Interface and the Delamination Tendency of Bilayer Tablets. J. Pharm. Sci..

[41] Newton J., Haririan I., Podczeck F. (2000). The influence of punch curvature on the mechanical properties of compacted powders. Powder Technol..

[42] Karalia D.S.A., Karalis V., Vlachou M. (2021). 3D-Printed Oral Dosage Forms: Mechanical Properties, Computational Approaches and Applications. Pharmaceutics.

