# Supplementary material for: Additive Manufacturing of Regorafenib Tablets: Formulation Strategies and Characterization for Colorectal Cancer
Source: Polymers (Basel). 2025 Aug 26;17(17):2302. doi: 10.3390/polym17172302 (PMC12431445; doi:10.3390/polym17172302)
Supplement: Supplementary file 1 [file polymers-17-02302-s001.zip › polymers-3767886-supplementary.pdf]

Supplementary information

Table S1. Calculations of the weight variation of the 3D-printed regorafenib.tablet

	Weight Variation									
**NO.**	1	2	3	4	5	6	7	8	9	10
**Weight(mg)**	1212.1	1127.2	1170.2	1202	1190.5	1188	1198.2	1158	1209.7	1175.8
**Mean**					1183.17					
**STD**					24.87					
**RSD**					2.10					

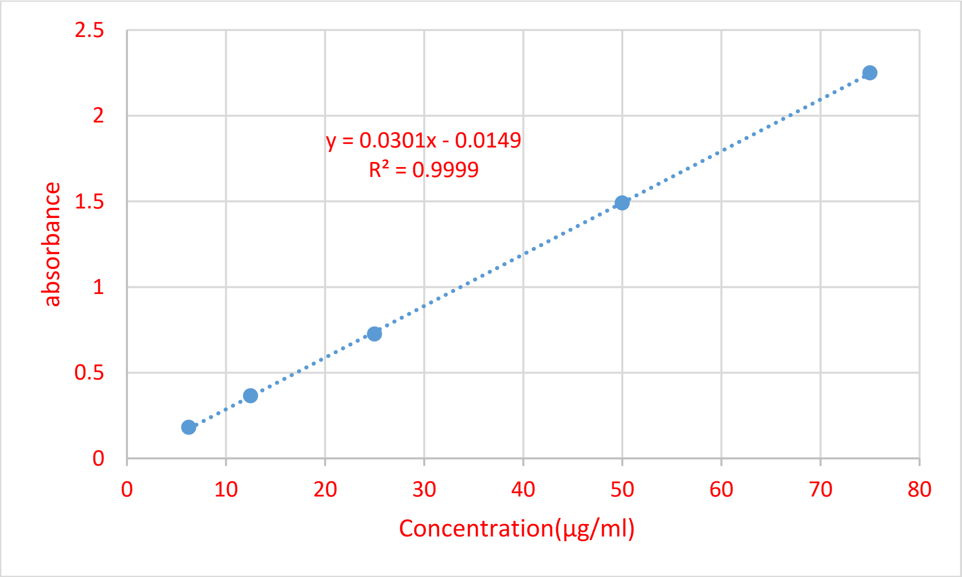

Figure S1. Calibration curve for release medium.
